# *GJB1* Mutation-A Disease Spectrum: Report of Case Series

**DOI:** 10.3389/fneur.2019.01406

**Published:** 2020-01-15

**Authors:** Jingwen Niu, Yi Dai, Mingsheng Liu, Yi Li, Qingyun Ding, Yuzhou Guan, Liying Cui, Liri Jin

**Affiliations:** Department of Neurology, Peking Union Medical College Hospital, Chinese Academy of Medical Sciences, Beijing, China

**Keywords:** X-linked Charcot-Marie-Tooth disease (CMTX1), *GJB1*, transient central nervous system symptoms, case series, peripheral neuropathy, central nervous system

## Abstract

**Introduction:** Patients with *GJB1* mutations manifested as pure central nervous system (CNS) involvement without peripheral neuropathy have not been adequately reported. To expand the disease spectrum of *GJB1* mutations, we report a case series.

**Methods:** Eleven patients from 9 families with *GJB1* mutations were reviewed. The clinical manifestations, electrophysiological studies, and gene tests were summarized.

**Results:** Nine patients had peripheral neuropathy, one patient had both peripheral neuropathy and mild cognitive impairment, and one patient had recurrent episodic limbs weakness and aphasia with normal electrophysiological study, indicating CNS involvement only.

**Discussion:** *GJB1* mutations form a clinical spectrum, including most patients with peripheral nerve involvement, those with both peripheral neuropathy and CNS involvement, and patients with CNS involvement only.

## Introduction

X-linked Charcot-Marie-Tooth disease type 1 (CMTX1), the second most common cause of hereditary neuropathy, is caused by mutations in *GJB1*, which codes for connexin 32 (Cx32). Cx32 is expressed in the myelinating Schwann cell of peripheral nerves, as well as the outer oligodendrocyte membranes in the central nervous system (CNS) (1). *GJB1* mutation usually causes a progressive sensorimotor neuropathy, CMTX1. Children and young adults with CMTX1 infrequently experience transient central nervous system manifestations (2, 3). We describe serial cases of *GJB1* mutation which form a clinical spectrum, including most patients with only peripheral nerve involvement, patient with both peripheral neuropathy and CNS involvement, and patient with only CNS involvement.

## Materials and Methods

Between 2015 and 2019, 11 patients from 9 families with *GJB1* mutations were retrospectively collected. The clinical manifestations, electrophysiological tests, and gene testing were reviewed. This study was carried out in accordance with the recommendations of the ethics committee of Peking Union Medical College Hospital with written informed consent from all subjects. For the publication of this article, written informed consent was obtained from subjects above the age of 16 and from parents for subjects under the age of 16. The protocol was approved by the ethics committee of Peking Union Medical College Hospital.

## Results

The clinical, electrophysiological, and genetic testing details of all 11 patients were listed in Table 1. Most patients were male (10/11), and they were young with a median age of 20 (ranging 6 to 34) at the time of investigation. Most patients had an onset of symptoms since early childhood.

**Table 1:** Characteristics of patients with *GJB1* mutation.

**No**.	**Age/sex**	**Onset age (years)**	**PNS manifestation**			**CNS manifestation**	**Edx**	***GJB1* Mutation**	**Family history**
			**Distal leg weakness**	**Pes cavus**	**Hand weakness**				
1	34/M	Early#	+	+	+	Mild cognitive impairment	D+A, M+S^*^	Val38Ala	Grandfather, a brother, two maternal aunts, and cousins
2	20/M	14	+	+	–	No	D+A, M+S	His123Tyr	None
3	26/F	24	+	+	–	No	D+A, M+S	Arg15Gln	Father and son
4	6/M	Early	+	–	–	No	D, M	Arg15Gln	His mother is patient 3
5	13/M	Early	+	+	–	No	D+A, M+S	Ser62Thr	Maternal grandfather. Mother has pes cavus without symptoms
6	21/M	19	+	+	+	No	D+A, M+S	Arg15Gln	Mother has pes cavus without symptoms
7	16/M	Early	+	+	–	No	D+A, M+S	Ile127Phe	A brother, mother, uncle, and grandmother on maternal side
8	23/M	Early	+	+	+	No	D+A, M+S	Ile127Phe	The patient is brother of patient 7
9	11/M	Early	+	–	–	No	D+A, M+S	Arg183his	Grandfather and cousin on maternal side
10	29/M	NA	+	–	–	No	NA	Ser26Leu	Maternal uncle
11	7/M	6	–	–	–	Episodic limbs weakness and dysphasia	Normal	Arg107Trp	None

* *D, demyelinating; A, axonal; M, motor; S, sensory*.

*#Early, early childhood*.

*PNS, peripheral nervous system; CNS, central nervous system; Edx, electrodiagnostic; NA, not available*.

Nine patients only had peripheral neuropathy, and the most common clinical manifestations were distal legs weakness and high foot arch. One patient (Patient 1) had both peripheral neuropathy and possibly CNS involvement manifested as mild cognitive impairment. One patient (Patient 11) had recurrent transient central nervous symptoms, with neither clinical nor electrophysiological evidence of peripheral neuropathy. The electrophysiological examine of most patients showed demyelinating and axonal polyneuropathy involving both motor and sensory nerves. Eight different mutations in *GJB1* were found, and 7 have been reported previously. The mutation of His123Tyr in patient 2 has not been reported in the literature. Most patients had a positive family history.

Patient 1 and patient 11, as the examples, are described in details as following.

**Figure 1:**
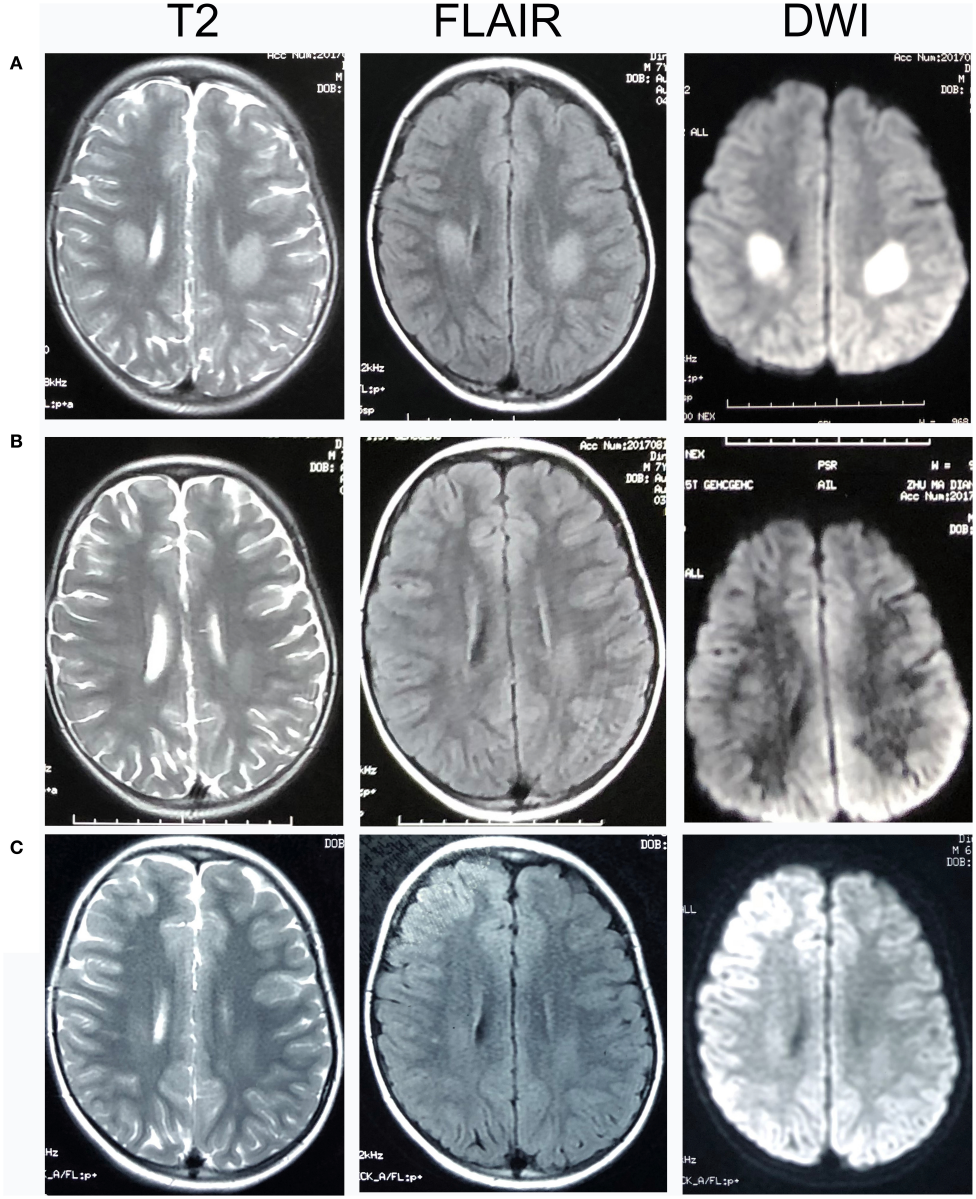
Head MRI of Patient 11. T2-, fluid-attenuated inversion recovery (FLAIR), and diffusion-weighted magnetic resonance imaging (DWI) images of Patient 11. **(A)** 2 days after the onset of his neurological syndrome; **(B)** 8 days after onset; **(C)** 1 month after onset. There were bilateral centrum semiovale T2 and DWI hyperintensities 2 days after onset, which significantly decreased on day 8 and at 1 month.

Patient 11 was a 7-year-old male, who had two clusters of episodic limbs weakness and dysphasia during the past 16 months. The first attacks occurred 16 months ago. During dinner, he suddenly had weakness of all four extremities and couldn't hold the spoon, and had difficulty in speaking. There was no alteration of consciousness. The symptoms resolved 2–3 min later but recurred for two times in similar fashion in the following 2 days. Provoking factors were denied, including fever/infection or traveling to high altitudes. Physical examination during the second episode showed right upper limb weakness and bilateral Babinski sign, which recovered several minutes later. The patient remained normal until 6 months ago, when he had another similar cluster of 3 attacks with episodic right hemiparesis and difficulty in speaking. Brain MRI performed within 2–3 days after the onset of each cluster showed bilateral centrum semiovale T2 and DWI hyperintensities, which significantly decreased 1 week later (Figure 1). No abnormality was found on brain MR angiography (MRA). At each cluster, serum electrolytes were normal. Glucose, protein, and cell counts in cerebrospinal fluid (CSF) were normal. Oligo-bands of both serum and CSF were positive. The organic acid and carnitine of blood and urine were within normal range. Tests for α-galactosidase, β-galactosidase, galactocerebrosidase, and arylsulfatase A in white blood cells were normal. Electroencephalography (EEG) was in the normal range for his age. Electromyography (EMG) and nerve conduction studies (NCS) performed in the local hospital, and those repeated 6 months later at our hospital were normal (Table 2). On admission to our hospital, a thorough neurological examination was normal, including normal deep tendon reflexes and muscular strength. Neither muscle atrophy nor pes cavus were found. DNA analysis showed a cytosine to thymine transition sequence alteration in the Cx32 allele of the *GJB1* gene in nucleotide position 391 predicting an arginine to tryptophane amino acid substitution at codon position 107. His mother had a heterogeneous mutation without any symptom or signs. This Arg107Trp mutation has been reported in CMTX family (4), and the mutant Cx32 protein has been proven to form defective junctional channels *in vitro* (5).

**Table 2:** Nerve conduction studies of patient 1 and patient 11.

**Nerve**	**Stimulation site**	**Distal latency (ms)**	**Amplitude (sensory in μV, motor in mV)**	**Velocity (m/s)**
**(1) Patient 1**				
**Sensory**				
Left median	1st finger		1.5 (21)	28.6 (46.7)
Left ulnar	5th finger		1.2 (7.1)	34.6 (46.6)
Right fibular	Ankle		NR (0.7)	
**Motor**				
Left median	Wrist	4.2 (3.8)	5.2 (9)	
	Elbow	10.8	2.5	32.1 (55)
	Axilla	13.7	2.1	38.7
Left ulnar	Wrist	3.4 (3)	10.2 (8)	
	Below elbow	6.9	7.8	34.0 (61)
	Above elbow	11.4	5.1	40.0 (50)
	Axilla	13.9	4.5	33.7
Right fibular	Ankle	NR (4.75)	NR (3.6)	
Right tibial	Ankle	7.2 (4.8)	0.5 (6)	
	Knee	26.3	0.3	20.8 (35.1)
Left fibular	Ankle	NR (4.75)	NR (3.6)	
**(2) PATIENT 11**				
**Sensory**				
Right median	1st finger		36	59.4
Right ulnar	5th finger		11	57.6
Left fibular	Ankle		3	64.9
**Motor**				
Right median	Wrist	2.6	15.3	
	Elbow	5.4	14.6	60.7
Left ulnar	Wrist	2.3	14.8	
	Below elbow	3.8	14.7	61.4
	Above elbow	5.4	15.2	67.9
	Axilla	6.5	14.9	58.0
Left fibular	Ankle	2.9	12	
	Fibular head	7.4	10.9	52.4
	Knee	8.3	10.9	64.5
Right tibial	Ankle	2.8	31.9	
	Knee	8.3	27.5	51.9

*Normal values indicated in parentheses. NR, no response*.

Patient 1, a 33-year-old male, complained of feet numbness for 2 years. Since childhood he had high-arched feet and was unable to dorsally extend his feet or toes. His maternal grandfather, two aunts, two cousins, and elder brother had similar symptoms of feet weakness and high-arched feet, and his mother had high-arched feet. Neurological examination showed areflexia in four limbs, mild muscular weakness and atrophy in hands, moderate muscular atrophy of distal lower limbs, bilateral pes cavus, and weakness of foot dorsiflexors. He had abolished vibratory sensation in distal limbs, and a steppage gait. Although he had no complaint of cognitive impairment, Mini-mental state examination (MMSE) and Montreal cognitive assessment (MOCA) scaled 26 and 24 (Visuospatial−1, language-1, executive function-2, memory-3, education years of 6+1), respectively. Blood glucose and serum immunofixation electrophoresis were normal. CSF glucose and cell counts were normal, with slightly elevated protein of 0.69 g/L. Brain MRI showed blurred symmetric bilateral hyperintensities of the periventricular white matter on T2 and fluid-attenuated inversion recovery (FLAIR) sequences. Electrophysiological studies showed abnormal mixed (demyelinating and axonal) polyneuropathy involving motor and sensory nerves (Table 2). DNA analysis showed a thymine to cytosine transition sequence alteration in the Cx32 allele of *GJB1* gene in nucleotide position 113 predicting a valine to alanine amino acid substitution at codon position 38.

## Discussion

It is well-established that in addition to the typical peripheral features of CMT, CNS involvement could be observed in patients with CMTX1. Previous studies showed that CNS manifestations do not appear to correlate with the stage and severity of peripheral neuropathy (6), and that recurrent CNS manifestations were reported in CMTX1 patients with initially subclinical peripheral neuropathy (7–9). Arayamparambil and Anilkumar (10) described a boy with no hyporeflexia or sensory impairments at the first episode of hemiparesis. However, NCSs were not performed to rule out subclinical peripheral neuropathy (10). Subclinical abnormalities of visual evoked responses (VERs) and brainstem auditory evoked responses (BAERs), and mild to moderate fixed abnormalities on neurological examination such as mild cognitive impairment, spasticity, or dysarthria and ataxia were also reported (11–13). Our case series of patients with *GJB1* mutation include typical CMTX1 patients with only peripheral neuropathy, patients with both peripheral and possibly CNS involvement, and interestingly, one patient manifesting only recurrent CNS symptoms and signs. To our knowledge, CNS manifestation without clinical or subclinical peripheral neuropathy in patients with *GJB1* mutation, though might has been seen by clinicians specializing in peripheral neuropathy, has not been adequately reported. These serial cases indicate that *GJB1* mutations might actually lead to a spectrum of disease, making a diagnosis more challenging due to genotypic and phenotypic variability.

*GJB1* encodes for Cx32, a protein subunit of intercellular channels found in gap junctions (GJs). Cx32 is widely expressed in several organs by many cell types, including Schwann cells and oligodendrocytes, the myelinating cells of the peripheral nervous system (PNS) and CNS, respectively (14). Six connexins oligomerize to form hemichannels, or connexons, and two connexons form gap junction channels that permit diffusion of ions and small molecules (1). In Schwann cells, Cx32 locates at the nodes of Ranvier and Schmidt-Lanterman incisures, while Cx26 and Cx43 are sparsely distributed in the plasma membrane of the cell body. Thus, no other connexins have yet been found to co-localize with Cx32 in the domains of non-compact myelin of adult Schwann cells. Cx32 mutations lead to either complete loss of function, or retaining but altered channel activity in Schwann cells, leading to peripheral neuropathy (14). In CNS, oligodendrocytes (Os) express Cx29, Cx32, and Cx47, while astrocytes (As) express Cx30 and Cx43. Cx32 forms homotypic and heterotypic channels, constituting O/O and A/O GJs. However, the O/O and A/O couplings are present in mice lacking Cx32. These suggest that Cx32 is not critical for CNS functioning, and that the subclinical and overt CNS manifestations of CMTX might be caused by a gain of function, rather than simple loss of function of Cx32 (11). Cx32 mutants might have trans-dominant-negative effects on Cx47, thereby reducing O/O and/or O/A coupling. Pro-inflammatory factors caused by trigger factors might directly inhibit glial GJs, exacerbating already tenuous GJ coupling and leading to clinical manifestations (11). These are possible explanations proposed for severe transient CNS dysfunction accompanied by white matter changes observed on MRI (6), as our patient 11. However, no known provoking factors were reported by his parents. Absence of provoking factors has been reported previously (2). It is possible that some unnoticed factors might have caused metabolic stress, thus disturbing unstable GJ coupling. Mild cognitive impairment, as in our patient 1, was also reported as CNS manifestations in CMTX patients (12). Patient 11 shows CNS symptoms and signs only and has no evidence of PNS involvement currently, representing an extreme form among the clinical spectrum of *GJB1* mutation. Tan et al. (4) reported patients with the same mutation as Patient 11, but they had peripheral neuropathy (4). According to literature, patients in one family with the same mutation in *GJB1* could have peripheral neuropathy with or without CNS symptoms (15). However, the reason why PNS was spared in Patient 11 is not well-understood. One possible explanation is that Patient 11 might probably develop the peripheral neuropathy in his later life. Anand et al. (9), Kim et al. (7), and Arayamparambil and Anilkumar (10) described patients with no clinical evidence of peripheral neuropathies at the first one or two CNS episodes, but clinical evidence of peripheral neuropathies appeared 2 months to 5 years later. However, these patients either had abnormal NCSs, indicating subclinical PNS involvement, or no NCSs at the first CNS episodes (7–9). There does not appear to be a “hotspot” for Cx32 mutations associated with transient CNS symptoms, and the CNS phenotypes in CMTX1 lack a consistent genotype-phenotype correlation (2).

The case of patient 11 reminds us that *GJB1* mutations should be a consideration in the differential diagnosis of recurrent reversible stroke-like CNS deficits in children, even without evidence of peripheral neuropathy. Stroke-like attacks with white matter lesions are rarely seen in children, and the common reasons include stroke, mitochondrial disease, acute disseminated encephalomyelitis (ADEM), and metabolic disorders (including Krabbe disease, Fabry disease, gangliosidosis, adrenoleukodystrophy, methylmalonicacidemia, etc). Even without evidence of peripheral neuropathy, *GJB1* mutation should be considered as a differentiation.

There were some limitations. There was only one single case with transient CNS symptoms and sparing of peripheral neuropathy, and the patient was young. We would follow up this patient to see if he would finally develop peripheral neuropathy. Also, we would explore if there were more such cases.

In conclusion, *GJB1* mutations actually represent a spectrum of disease, including patients with either peripheral neuropathy or CNS involvement, or both. *GJB1* mutations should be a consideration in the differential diagnosis of recurrent reversible stroke-like symptoms in children.

## Data Availability Statement

All datasets generated for this study are included in the article/supplementary material.

## Ethics Statement

The studies involving human participants were reviewed and approved by This study was carried out in accordance with the recommendations of the ethics committee of Peking Union Medical College Hospital with written informed consent from all subjects. All subjects gave written informed consent in accordance with the Declaration of Helsinki. The protocol was approved by the ethics committee of Peking Union Medical College Hospital. The patients/participants provided their written informed consent to participate in this study.

## Author Contributions

JN study design and conceptualization, data analysis, and manuscript drafting. YD study design and conceptualization, data collection, and analysis, manuscript revision. ML data collection, manuscript revision. YL and QD data collection and analysis. YG and LC manuscript revision. LJ study design, manuscript drafting, and revision.

### Conflict of Interest

The authors declare that the research was conducted in the absence of any commercial or financial relationships that could be construed as a potential conflict of interest.

## Abbreviations

ADEM: acute disseminated encephalomyelitis
As: astrocytes
CNS: central nervous system
CMTX1: X-linked Charcot-Marie-Tooth disease type 1
CSF: cerebrospinal fluid
Cx32: connexin 32
EEG: electroencephalography
EMG: electromyography
FLAIR: fluid-attenuated inversion recovery
GJ: gap junction
MRA: MR angiography
MMSE: Mini-mental state examination
MOCA: Montreal cognitive assessment
NCS: nerve conduction studies
Os: oligodendrocytes
PNS: peripheral nervous system
